# *checkCIF* validation ALERTS: what they mean and how to respond

**DOI:** 10.1107/S2056989019016244

**Published:** 2020-01-01

**Authors:** Anthony L. Spek

**Affiliations:** a Crystal and Structural Chemistry, Bijvoet Center for Biomolecular Research, Utrecht University, Padualaan 8, 3584CH Utrecht, The Netherlands

**Keywords:** *checkCIF*, validation alerts, *PLATON*, crystal structure.

## Abstract

Authors of a paper that includes a new crystal-structure determination are expected to not only report the structural results of inter­est and their inter­pretation, but are also expected to archive in computer-readable CIF format the experimental data on which the crystal-structure analysis is based. Additionally, an IUCr/*checkCIF* validation report will be required for the review of a submitted paper. Such a validation report, automatically created from the deposited CIF file, lists as ALERTS not only potential errors or unusual findings, but also suggestions for improvement along with inter­esting information on the structure at hand. Major ALERTS for issues are expected to have been acted on already before the submission for publication or discussed in the associated paper and/or commented on in the CIF file. In addition, referees, readers and users of the data should be able to make their own judgment and inter­pretation of the underlying experimental data or perform their own calculations with the archived data. All the above is consistent with the FAIR (findable, accessible, inter­operable, and reusable) initiative [Helliwell (2019 ▸). *Struct. Dyn.* **6**, 05430]. Validation can also be helpful for less experienced authors in pointing to and avoiding of crystal-structure determination and inter­pretation pitfalls. The IUCr web-based *checkCIF* server provides such a validation report, based on data uploaded in CIF format. Alternatively, a locally installable *checkCIF* version is available to be used iteratively during the structure-determination process. ALERTS come mostly as short single-line messages. There is also a short explanation of the ALERTS available through the IUCr web server or with the locally installed *PLATON*/*checkCIF* version. This paper provides additional background information on the *checkCIF* procedure and additional details for a number of ALERTS along with options for how to act on them.

## Abstract 2

This paper provides additional background information on the *checkCIF* procedure and additional details for a number of ALERTS along with options for how to act on them.

## Introduction   

Developments in diffractometer technology and software have made the collection of X-ray diffraction data on single crystals rapid and routine. The same applies in most cases for the preliminary structure-solution phase, given diffraction data of reasonable quality. Current structure-solution programs such as *SHELXT* (Sheldrick, 2015*a* ▸), *SUPERFLIP* (Palatinus & Chapuis, 2007 ▸) and *SIR* (Burla *et al.*, 2003 ▸) have made the preliminary solution of the phase problem in most cases a trivial black-box operation. The bottleneck to a publishable report is often the final analysis, validation, reporting and proper archiving of the experimental data and refinement results. The large volume of automated ‘routine’ crystal-structure determinations often leaves less time available for a detailed analysis of the results. Unfortunately, it is easy for less crystallographically trained authors, depending on the automated procedures, to fall into one of the well-known pitfalls such as pseudo-symmetry (Clegg, 2019 ▸). The reported chemistry may be wrong when signals such as short inter­molecular contacts, that are clear for experienced investigators, are misunderstood or ignored.

Not all published crystal-structure studies are based on the best attainable diffraction data, but rather on data that are considered to be suitable for the purpose of the study (Thompson, 2019 ▸). A good-looking ‘*ORTEP*’ illustration in a chemical journal is often considered as sufficient proof of the reported chemistry. Crystal structures are not always ‘well-behaved’, often related to sub-optimal crystal quality, experimental issues, or disorder or twinning. Structural disorder can be pursued in great detail with constraints and restraints or handled in a way sufficient for the study of the main species of inter­est, for example using tools such as *PLATON*/SQUEEZE (Spek, 2015 ▸) for handling disordered solvents as part of the structure refinement. Readers need to know whether reported geometrical details, such as inter-mol­ecular contacts or bond distances, involve restrained or constrained parameters. All this and residual issues should be clearly documented in the structure report.

Reported crystal structures end up in the Cambridge Structural Database (CSD; Groom *et al.*, 2016 ▸) as curated but otherwise largely unqualified database entries. It is therefore of the utmost importance that all experimental data, data reduction and refinement details are archived as well. In that way, users of those data can do their own evaluation of the reported crystal structures or use the experimental data for more detailed studies in a different context. Those data may be hard to obtain again, either because of the involved synthesis of the compound of inter­est or the difficulty in obtaining a particular polymorph again.

During the 1980s, with the growth in the number of structure reports, the need for a standard computer-readable format for the exchange and archival of crystallographic data became clear. Obviously, that would avoid the error-prone retyping of numerical information, such as the refined parameter values, into the desired format from typo-prone manuscripts. It would also make the diffraction data more accessible for processing. Such a standard, called CIF, was proposed by Hall *et al.* (1991 ▸). One of the early adopters of that format was George Sheldrick with his new and popular 1997 version of the *SHELXL* (Sheldrick, 2008 ▸) structure-refinement program, making CIF widely accepted as a data-archival format, along with the CIF-style structured FCF file with observed and calculated reflection data. Those data were previously available only as printed *F*_obs_, *F*_calc_ and σ(*F*_obs_) tables. Inter­estingly, *SHELXL* does not read and write CIFs for its own input and output. For efficiency, it uses for its calculations the non-CIF-structured RES and HKL computer- and human-readable files for the input and storage of refinement details and reflection data, respectively. Those files have also become a *de facto* exchange standard for a number of applications and programs such as the CSD utilities (Groom *et al.*, 2016 ▸). Today, the archived material is expected to include not only the structural results, but also the unmerged reflection data (either as a CIF-structured reflection-data loop or as embedded *SHELXL-*style *name.hkl* file) and details of the data reduction and non-standard refinement details (CIF-structured or as an embedded *SHELXL-*style *name.res* file). Those files can easily be extracted from the CIF with the *shredcif* utility and used for alternative refinements. Most current structure-refinement packages include that information in the CIF file.

The introduction of the CIF standard also opened the way for the automated checking of the archived data for their inter­nal consistency and completeness, which was needed to handle the exploding number of structure reports. The Inter­national Union of Crystallography (IUCr) journal *Acta Crystallo­graphica Section C* pioneered automated structure validation as a tool for authors, referees and readers. This project started with flagging missing data and inconsistencies and testing numerical data against expected values. The result was a report consisting of a list of ALERTS, with associated A, B and C levels of importance, for issues that needed to be addressed. It should be clear that ALERTS are not necessarily errors. They might also point to inter­esting features in a crystal structure. G-level ALERTS, which were introduced later, are mostly informative but are not to be neglected. All ALERTS should be checked by the authors: a set of lower-level ALERTS may in combination point to a serious issue that needs to be addressed.

Currently, IUCr/*checkCIF* includes, in addition to the above-mentioned ALERTS, *PLATON*/*checkCIF* (Spek, 2003 ▸) based ALERTS (starting with ‘PLAT’ followed by a number) implementing some 500 additional tests for issues such as missed symmetry, missed twinning and missed solvent-accessible voids in the crystal structure. This paper is a follow-up to earlier papers on the validation issue and *checkCIF* (Spek, 2003 ▸, 2009 ▸, 2018 ▸). It offers additional background details for a number of more commonly issued ALERTS along with suggestions on how to address them.

Three types of archived information should be available: (*a*) crystal data such as cell parameters, symmetry, refined model parameters, derived geometry and *R* values – those data (*name.cif*) are checked for completeness, inter­nal consistency and expected parameter values; (*b*) the final ‘FCF’ file (*name.fcf*) with calculated and observed intensities and associated σ(*I*)/weight to check the reported final *R* and *S* values along with an analysis-of-variance analysis of the refinement results; (*c*) the unmerged *h*, *k*, *l*, *I*_obs_, σ(*I*_obs_) reflection data [CIFs created using *SHELXL* (Sheldrick, 2015*b* ▸) include this ‘HKL’ file (*name.hkl*) along with the final refinement instruction ‘RES’ file (*name.res*) as embedded data as values of the *_shelx_hkl_file* and *_shelx_res_file* CIF datanames along with associated checksums reported as values of the datanames *_shelx_hkl_checksum* and *_shelx_res_checksum*, respectively]. No archival of the final FCF file is needed in the case of matching calculated and reported checksums for the *name.res* and *name.hkl* files. In the case where a final *name.fcf* is recreated with *SHELXL* from the embedded *name.hkl* and *name.res* files and recalculated, the final *R* and *S* values are compared with those reported in the CIF for consistency. Otherwise the final *name.fcf* file should be archived as well.

The alternative datanames *_iucr_refine_instructions_details*, *_iucr_refine_reflections_details* and *_iucr_refine_fcf_details* are available to embed refinement and unmerged HKL and FCF data associated with non-*SHELXL*-based refinement programs. *OLEX2* (Dolomanov *et al.*, 2009 ▸) offers both a refinement based on *SHELXL* and a *SHELXL-*compatible refinement tool (REFINE). In the former case, standard *SHELXL name.cif* and *name.fcf* files are created. In the latter case, the unmerged reflections are included in the CIF with a looped structure and the *name.fcf* file embedded in the *_iucr_refine_fcf_details* field. The *CRYSTALS* (Betteridge *et al.*, 2003 ▸), *JANA* (Petricek & Dusek, 2000 ▸) and *WinGX* (Farrugia, 2012 ▸) refinement packages follow similar archival schemes. *A Guide to CIF for Authors* is available at https://www.iucr.org/__data/assets/pdf_file/0019/22618/cifguide.pdf.

## Experimental data and data reduction   

Most current crystal structure reports are based on data collected with area detectors as opposed to previously used serial detectors. Some of the ALERTS and validation criteria have their origin in the use of serial detectors. They are kept for backward compatibility. Many non-applicable ALERTS are suppressed automatically when obvious from the other information available in the CIF.

### Experimental data   

It is good practice to collect diffraction data up to at least *copper sphere* resolution, *i.e.* sin (θ)/λ = 1/1.5418 = 0.65 Å^−1^, in order to achieve a good data-to-refined-parameter ratio. That resolution corresponds to 27.5° in θ for Mo *Kα* radiation and a real space resolution of 0.77 Å, *i.e*. about half of the C—C bond distance. The reflection data set is expected to be essentially complete and have a high redundancy (*i.e.* multiple-measured data). The latter allows for the calculation of the *R*_int_ value as a measure of the inter­nal consistency of similar intensity measurements and their use as a basis for multi-scan-based correction for absorption. Sometimes, the above resolution can only be achieved, with a significant fraction of reflections above the noise level, when data are collected at low temperature. Unfortunately, physical restrictions such as data collection with a Cu *Kα* X-ray source, will allow data to be collected only up to a resolution of about 0.6 Å^−1^. For that reason, data sets are expected to be at least complete up to that value (*i.e.* ∼25° in θ for Mo *K*α). This does not imply that meaningful data should be removed from the structure analysis beyond that resolution without a valid reason. Arguments based on limited available instrument time do not count anymore with currently used area detectors. A sensible cut-off value might be a value beyond which there is only noise, not when there is still a significant number of reflections with *I* > 2σ(*I*) at the applied cut-off. A poor data-collection strategy may lead to a cusp of missing reflections. Randomly missing reflections, *e.g*. due to overflows, may be acceptable for the least-squares structure refinement. Unfortunately, incomplete data sets may be unavoidable in case of data collection on a low-symmetry crystal in a high-pressure cell. Particular attention is needed for the low-order reflections when difference-density maps are of inter­est. Extra efforts, such as a special scan with the detector further away, should be made to include them or remove them from the data set when (partly) obscured by the beamstop. Low-order reflections may carry significant information.

It might be helpful to make a record of special features encountered during diffraction image processing such as un-indexed (weak) spots, streaks, diffuse scattering, and split-up or broadened reflection profiles. They might provide a clue to the reason why or when problems arise during structure determination and refinement.

Unit-cell dimensions, as all measured and derived qu­anti­ties, are to be accompanied with a standard uncertainty. They are commonly based on a least-squares treatment of the setting angles of a number of reflections. With serial detectors, that number was traditionally around 25 whereas with area-detectors, approximate setting angles of many thousands of reflections are used. Standard uncertainties tend to be much smaller in the latter case and often unrealistic when confronted with the variance in the cell dimensions obtained in unit-cell dimensions for crystals from the same or different batches. It is likely best to accept and report standard uncertainties as they come out of the least-squares-fit program and report them along with the number of reflections involved and the θ range. Consider them as a measure of inter­nal consistency and do not arbitrarily apply an empirical and undocumented multiplier to them to obtain supposedly realistic values. Otherwise, users of those data might unknowingly apply yet another multiplier. Errors in cell dimensions and their standard uncertainties may have significant effects on the reported derived geometry values. The reported wavelength should have sufficient significant figures. Their values are directly related to the wavelength value used in the calculation of the cell parameters.

### Data reduction   

#### Absorption correction   

Given a crystal with faces that can be indexed and bathed fully in the homogeneous part of an X-ray beam, there are numerical and analytical ways to correct for absorption. This ideal is rarely achieved in current practice. Crystals are rarely bathed homogeneously, as assumed by the above correction algorithms. Instead, the experimental multi-scan technique is used where an absorption surface is constructed using the intensity differences between supposedly identical reflections (either symmetry related or measured on different orientations about the diffraction vector). It is important to report the actual correction range and compare that range with the range expected based on the crystal dimensions. In addition, a significant reflection redundancy is in order for a meaningful correction. Sometimes, more than one absorption correction is concatenated (*e.g*. numerical or analytical correction followed by multi-scan-based correction). This should be reported in the CIF in the special details section.

#### Symmetry   

The symmetry of the electron density of a crystal structure can, in the majority of reported cases, be associated with one of the 230 distinct three-dimensional symmetry patterns. Each of those patterns has a Hermann–Maughuin symbol associated with it, *e.g. P*2_1_/*c*. Those symbols imply a standard unit cell and origin choice setting, in this case at the inversion center, along with a twofold screw axis and a *c*-glide. Different choices include *P*2_1_/*a* and *P*2_1_/*n* based on a different choice of the unit cell or even with the origin off the inversion center. Even *P*2_1_/*b* is possible if the unique axis is taken to be *a* or *c*. There can be various reasons not to report a structure in the standard setting. Sometimes it is considered beneficial to select the *P*2_1_/*n* setting rather than *P*2_1_/*c* because that unit cell has a closer to 90° β angle, which leads to better convergence and lower correlations between the refined atomic parameters (Feast *et al.*, 2009 ▸). In addition, a non-standard choice may be useful when its relation with another structure is to be shown (*e.g.* a redetermination or a phase transition).

Fortunately, the symmetry of a structure, needed for the various computations, is completely described by the set of symmetry operators or the subset of space-group generators that can be expanded into the full set. Those symmetry operators are not easily or uniquely derivable from the Hermann–Maughuin symbols. The alternative Hall symbol (Hall, 1981 ▸) has been introduced to record the essential space-group symmetry generators in one symbol and suitable to be used to uniquely derive the full set of symmetry operators from them. As an example, the Hall symbol for *P*2_1_/*n* with the origin on the inversion center is –*P* 2*yn*, where ‘−’ represents the inversion operation −*x*, −*y*, −*z* and ‘2*yn*’ the screw axis operation 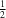 − *x*, 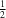 + *y*, 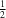 − *z*. The two symmetry operations are sufficient to create the complete set of symmetry operations of the space group. It should be noted that Hall symbols are not necessarily unique. Different choices of generators may lead to the same set of symmetry operators. Hall symbols can be especially useful as a space-group-symmetry identifier in the case of a non-standard setting where no Hermann–Maughuin symbol has been officially defined. A CIF is expected to report both the Hermann–Maughuin and Hall symbols along with the full set of symmetry operations. *checkCIF* checks for the presence of those data along with their mutual consistency and their consistency with the reported crystal system. It currently ignores the inconsistency between Hermann–Maughuin and Hall symbols in cases of uncommon non-standard settings. Recent refinement programs such as *SHELXL2018* (Sheldrick, 2015*b* ▸) and later include proper Hall symbols in the CIF for standard space-group settings. *PLATON*/*checkCIF* (Spek, 2003 ▸) will propose in many cases a Hall symbol consistent with the set of supplied symmetry operations to be inserted in the CIF. In other cases, the Hall (1981 ▸) paper will give guidance to create a proper Hall symbol. The current *checkCIF* tool cannot handle symmetries of non-three-dimensional structures such as those for incommensurate structures.

## Structure solution   

The path from a preliminary structure to a refined structure is full of pitfalls for the unwary. Validation can be helpful in that process and should not be postponed to the publication stage where it takes much more effort to address problems with a structure, in particular when a new data collection will be needed. Either the IUCr *checkCIF* server or a locally installed version of the program *PLATON*/*checkCIF* can be used for that. *PLATON* also includes additional utilities to investigate ALERTed issues such as hydrogen-atom location, twinning and ADDSYM reported issues.

### Connected set   

Preliminary structure solution does not always result in a logically ordered, sensibly labelled and connected set of atoms in the asymmetric unit. That is often not essential at the refinement stage but is certainly a requirement for a meaningful and professional report. It is good practice to make sure that all species in the asymmetric unit have their center-of-gravity within the unit-cell bounds. Exceptions include small species such as water mol­ecules and counter-ions. Those are best located near their inter­action points with the main species. This limits the number of symmetry operations needed in hydrogen-bond tables and offers a cleaner presentation.

Symmetry operations on coordinates are generally represented by a code of the type **s_uvw**, where **s** refers to the sequence number of the symmetry operation in the previously defined list of symmetry operators in the CIF and **u**, **v** and **w** for unit-cell translations. The symmetry code for the primary species is represented by 1_555 and that for a species moved by two units in the *a*-axis direction and one unit in the −*c*-axis direction by 1_754. This way of representation of symmetry codes has its origin in a similar translation encoding used in the mol­ecular graphics program *ORTEP* (Johnson, 1976 ▸) with its origin in the 1960s. The main drawback of this type of encoding is that only translations in the range −5 to 4 can be represented with this code. This can be a problem with polymeric structures and long mol­ecules that stretch over multiple unit cells. A proper use of the location of a species within the unit-cell bounds, close to the origin, might be essential in those cases. Care should be taken when, for example, hydrogen-bond table information is copied from external programs such as *PLATON*. The symmetry codes and sequence must be identical to those defined in the CIF.

### Missed symmetry   

The applicable symmetry of a crystal structure is not always obvious. It depends on issues such as the radiation used in the experiment. Neutrons may see a different symmetry than X-rays. The former may ‘see’ magnetic spin-structure symmetry where the latter just indicate electron-density symmetry. The majority of X-ray structure reports are based on the space- and time-averaged electron density with a three-dimensional translation lattice, corresponding to sampling of the diffraction data at discrete points in diffraction (reciprocal) space. All diffraction outside those discrete points with possible information on order/disorder, superlattices and incommensurate behavior is then ignored.

All three-dimensional structures can be described in space group *P*1. Some currently used structure-solution programs such as *SHELXT* do just that and search for higher symmetry at a later stage. In practice, symmetry relations, compatible with the translation lattice, can be found between atomic positions within a certain tolerance. Collectively, they form the applicable space group. Alternatively, or in support, similar relations can be found in reciprocal space (as implemented in *SIR* and *SHELXT*).

*checkCIF* uses the *PLATON*/ADDSYM routine, an extended implementation of the MISSYM algorithm (Le Page, 1987 ▸, 1988 ▸) to check the reported space-group symmetry or to propose a possible higher-symmetry description within default tolerance settings. Those defaults are set at values needed to catch both higher proposed symmetry with poor data sets or inter­esting pseudo-symmetry. For that reason, hydrogen atoms and disordered atoms are ignored in the ADDSYM analysis. Also, the difference in atom types is ignored in order to catch atom-type mis-assignments (carbon for nitrogen, *etc*.). In addition, a certain percentage of atoms is allowed not to conform with the proposed higher symmetry. For that reason, *checkCIF* space-group ALERTS should always be checked, *e.g.* with the *PLATON*/ADDSYM tool using different tolerance settings. Examples of missed symmetry can be found in Marsh & Spek (2001 ▸).

The set of coordinates tested by ADDSYM for higher symmetry necessarily represents only part of the information present in the experimental reflection data. Cases of pseudo-symmetry need detailed inspection of the reflection data, including significant intensity values for supposedly systematic extinctions. Pseudo-symmetry easily comes together with twinning. In those cases, the purely coordinate-based ADDSYM algorithm needs to be supplemented by or alternatively tested with the reflection data. Good examples for that are described in detail by Clegg (2019 ▸). Eventually, refinement based on the proposed higher-symmetry options should provide the final verdict. Sometimes, even the diffraction data do not carry the necessary information to make a final decision between *e.g. P*1 and *P* 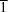 in the absence of knowledge of enanti­opurity of the crystal.

### Twinning detection   

*checkCIF* includes a test for twinning. Both missed and possibly already applied but unreported twinning is suggested, along with an estimated twinning fraction and estimated *R*-value drop when applied. Reported cases of twinning can be non-merohedral, pseudo-merohedral and merohedral. The first two should already be indicated during the image-processing and data-reduction stage as additional or extended diffraction spots. Merohedral twinning might only show up during the structure solution and refinement stage with unexpectedly high *R* values or apparent systematic absences not consistent with any known space group.

The routine used in *checkCIF*, TwinRotMat, is based on the observation that twinning results in a significant number of reflections with *I*_obs_ >> *I*_calc_ for the untwinned model refinement. Those reflections are overlapped by a strong reflection with (approximately) the same diffraction θ angle from a rotation-related twin-component lattice. Analysis of multiple such cases may lead to proposed candidates for the twinning operation at hand. Twinning detection with TwinRotMat may obviously be hampered when applied to reflection files where all strongly deviating reflections have already been removed from the refinement.

The TwinRotMat tool in a local implementation of the program *PLATON* can be used for more detailed analysis of the suggested twinning operations. Optionally, an HKLF5-style reflection file can be created for handling non-mero­hedral twinning with the *SHELXL* refinement program. Unfortunately, for the creation of such a file, empirical criteria have to be applied concerning overlapping reflections. In many cases, it is therefore often better to create HKLF5 files at the image-integration stage.

## Refinement   

The experimental electron density in a crystal structure is approximated by a set of structure model parameters. The most commonly used form is the atom-in-mol­ecule (AIM) model, assuming spherical atomic scattering factors. The parameter set includes the positional parameters of the atoms, the anisotropic displacement parameters on the non-hydrogen atoms and isotropic displacement parameters for the hydrogen atoms. Some additional parameters are experiment-related such as the scaling factor of the observed diffraction data and correction factors for twinning and extinction. More involved models with non-spherical scattering factors, anharmonic displacement parameters or incommensurate structure models will not be considered here.

### Constraints and restraints   

Most of the model parameters can be refined. An exception involves space groups with a floating origin such as *P*1 and *P*2_1_. That situation is usually handled by either fixing one or more of the positional parameters of one of the atoms, or better, limiting the shift of the center-of-gravity of the atoms or similar. There are also symmetry restrictions on refineable coordinates and anisotropic displacement parameters of atoms lying on special positions. Most current structure-refinement programs handle those issues automatically.

Additional parameter restrictions may be needed, subject to the resolution, the reflection data-to-parameter ratio or in the case of disorder in the structure. Restrictions come in two varieties (Müller *et al.*, 2006 ▸). Constraints fix parameters to a fixed value or to the value of another (refineable) parameter. Restraints (sometimes confusingly called ‘soft constraints’) are ‘springs’ of variable strength to draw parameters to desired parameter values, subject to the counter force from the experimental data.

Common restrictions include the fixing of carbon-bonded hydrogen atoms on calculated positions and riding them on their carrier atom in the refinement. This will give a better data-to-parameter ratio and avoids hydrogen atoms that refine to unrealistic distances due to noisy diffraction data. The positions of hydrogen atoms bonded to non-carbon atoms are often less predictable because their location depends on the presence of available acceptors in their environment. Their positions should be confirmed in a difference-density map (*vide infra*) and refined whenever possible as proof of their validity (Fábry, 2018 ▸).

Disorder resulting in overlapping atomic sites will require the application of constraints and/or restraints in order to keep the refinement stable and the model realistic.

The application of satisfactory constraints and restraints to model part of a structure may result in a more reliable model of the ordered part of inter­est of a structure. This applies in particular for the refinement of target mol­ecules along with only weakly inter­acting disordered solvents. However, when the inter­est lies in the disordered part of a structure, there is always the question about what additional information is obtained beyond what is already known or included explicitly; see also Immirzi (2009 ▸) and Spek (2018 ▸).

Some refinement programs include parameter value shift-limiting constraints or restraints. *SHELXL* includes the DAMP instruction to control extreme parameter shifts of refined parameters that are poorly defined by the reflection data. This tool might be useful in the preliminary stages of the structure refinement but should be removed from the final refinement cycles and replaced by fixing still troublesome parameters to reasonable values. A strong damping factor may erroneously give the illusion of a converged structure with unreasonably low s.u.’s on the refined parameters.

All applied constraints and/or restraints should be reported in the experimental section and explained in the discussion sections of a paper.

### SQUEEZE   

An alternative way to address the disordered-solvent problem is the use of the SQUEEZE algorithm. With that technique the solvent-accessible volume in a structure is identified and used to calculate the contribution of the scattering material found in that volume to the total calculated structure factor (Spek, 2015 ▸). The exact content of the disordered solvent volume is not determined explicitly with this method, but can sometimes be guessed from the reported total electron count of the integrated density in that volume, along with information about the solvents used during the synthesis and crystallization. The quality of the reported electron count is dependent on the quality, resolution and completeness of the diffraction data and the way the ordered part of the structure is modelled satisfactorily. Improper handling of low-angle data or omitting those data may seriously hamper the usefulness of the electron count. Disordered solvents contribute in particular to low-angle data. The missing data are estimated, but that is only partly satisfactorily. Inclusion of reflections that are hampered by the beamstop can pose a serious problem.

The disordered solvent contribution to the calculated structure factors as obtained with the current implementation of the SQUEEZE algorithm does not include resonant scattering contributions for *e.g.* disordered CHCl_3_, making the absolute-structure determination of a light-atom main mol­ecule of inter­est less reliable. A workaround was reported by Cooper *et al.* (2017 ▸).

The SQUEEZE tool is implemented in *PLATON*. Relevant details of the SQUEEZE treatment are included at the end of the *name.cif* embedded *name.fab* file. An alternative, and slightly different, implementation of the SQUEEZE concept is available in the *OLEX2* software (Dolomanov *et al.*, 2009 ▸).

### Difference density maps   

**Figure 1:**
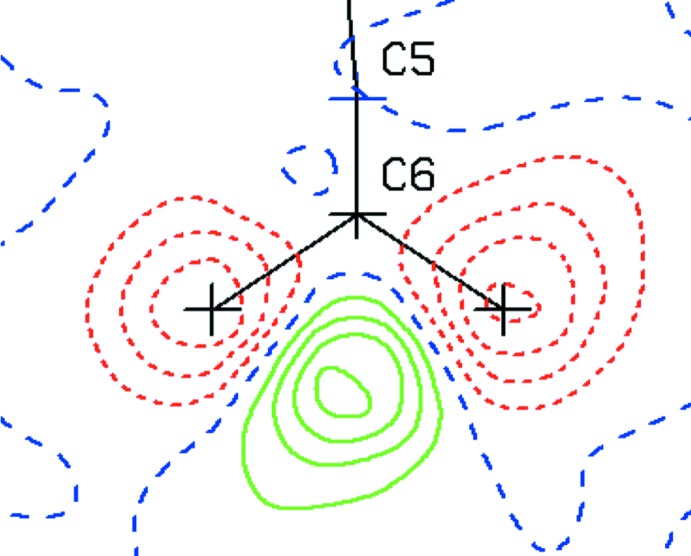
Example of hydrogen atoms wrongly calculated in *sp* ^2^ rather than *sp* geometry positions. Wrongly calculated hydrogen-atom positions show up as negative (red) densities in the difference density map and correct places as positive (green) densities. Contours are drawn with 0.1 e Å^−3^ inter­vals.

**Figure 2:**
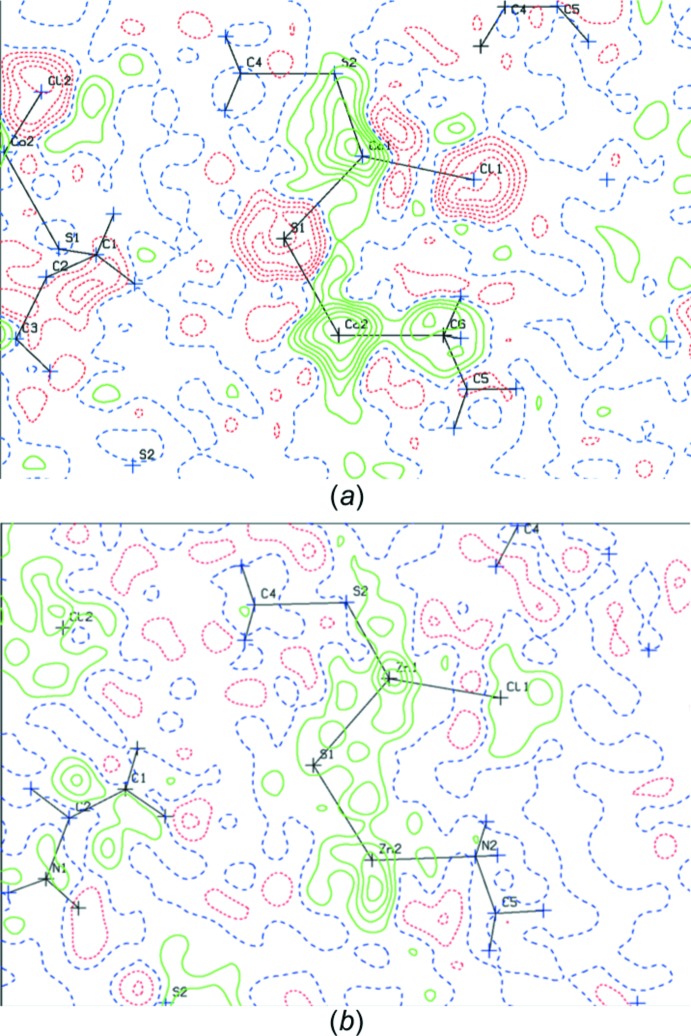
(*a*) Difference density map for a structure with wrong atom-type assignments. (*b*) Difference map with correct atom-type assignments. Contours are drawn with 0.1 e Å^−3^ inter­vals.

An *F*_calc_-phased |*F*_obs_| − *F*_calc_ difference-density map offers an extremely useful tool to detect problems with a structure determination. Such a map should be close to featureless with similar positive and negative density excursions of less than ∼±0.5 e Å^−3^. It might show regions of positive density about 1 Å from light atoms such as carbon, nitro­gen and oxygen, generally an indication for a missed hydrogen-atom site. Negative densities on a calculated hydrogen-atom site may indicate misplaced hydrogen atoms, *i.e.* a location where there is no density to be modelled. A common problem are the hydrogen-atom positions on a C—CH_3_ fragment that often need to be rotated by 60° around the C—C bond from their current calculated positions. The difference map section through the three hydrogen-atom sites will then show six alternating negative and positive density minima and maxima. A wrong hybridization instruction for the calculation of the hydrogen-atom positions may show up as shown in Fig. 1 ▸. Other examples are ring substituents where C—H and N sites have to be inter­changed and hydrogen atoms on N atoms that are calculated on planar *sp* ^2^ locations rather than with tetra­hedral *sp* ^3^ geometry. Positive or negative densities on non-hydrogen-atom sites may indicate wrong atom-type assignments (Fig. 2 ▸). Validation ALERTS will generally point to those problems. Thoughtful examination of the model (and possible consultation with synthetic chemists) will help to resolve them.

**Figure 3:**
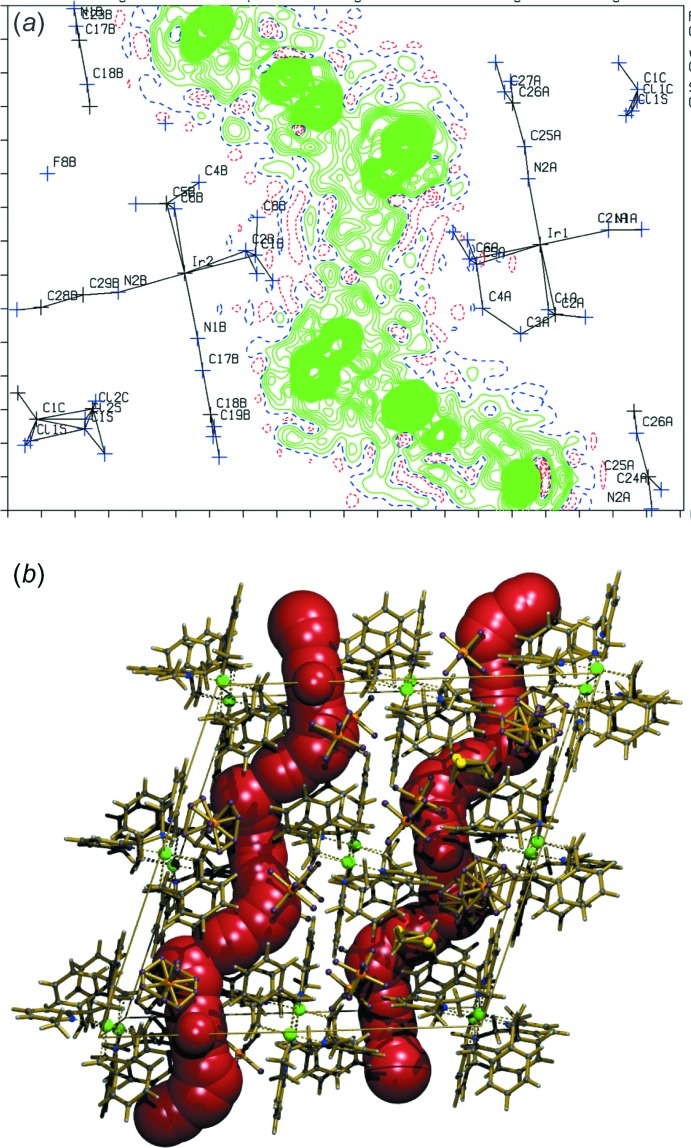
(*a*) Example of a structure with infinite channels filled with unresolved residual density and (*b*) display of the infinite channel, artificially filled with spheres.

Other issues in the difference-density map may easily escape attention or be explained away as being due to poor data when one just relies on the maximum and minimum residual density excursions reported by the refinement software. Some programs do not report difference-map density on atom sites. Contoured two- and three-dimensional difference-density maps offer a more detailed perspective. Significant approximately spherical density in a difference-density map cannot be ignored. Such density is usually inter­pretable as artificial (due to wrong assumed symmetry) or real structural disorder, substitutional disorder (*e.g*. a mixture of Cl and CH_3_ substituents) or missed twinning. Disordered solvent sites, in particular when occupied by a mixture of solvents or solvents (incommensurately ordered) in infinite channels (Fig. 3 ▸), may be missed when one relies on peak-search algorithms that assume ellipsoidal density shapes, *e.g*. density ridges are easily missed. Algorithms, such as the one used in *checkCIF*, are available to detect those regions in the structure as solvent-accessible voids. The SQUEEZE algorithm (van der Sluis & Spek, 1990 ▸; Spek, 2015 ▸) can be used to both investigate the content of those voids and, in the case where significant density is found, used to take blurred positive density into account.

**Figure 4:**
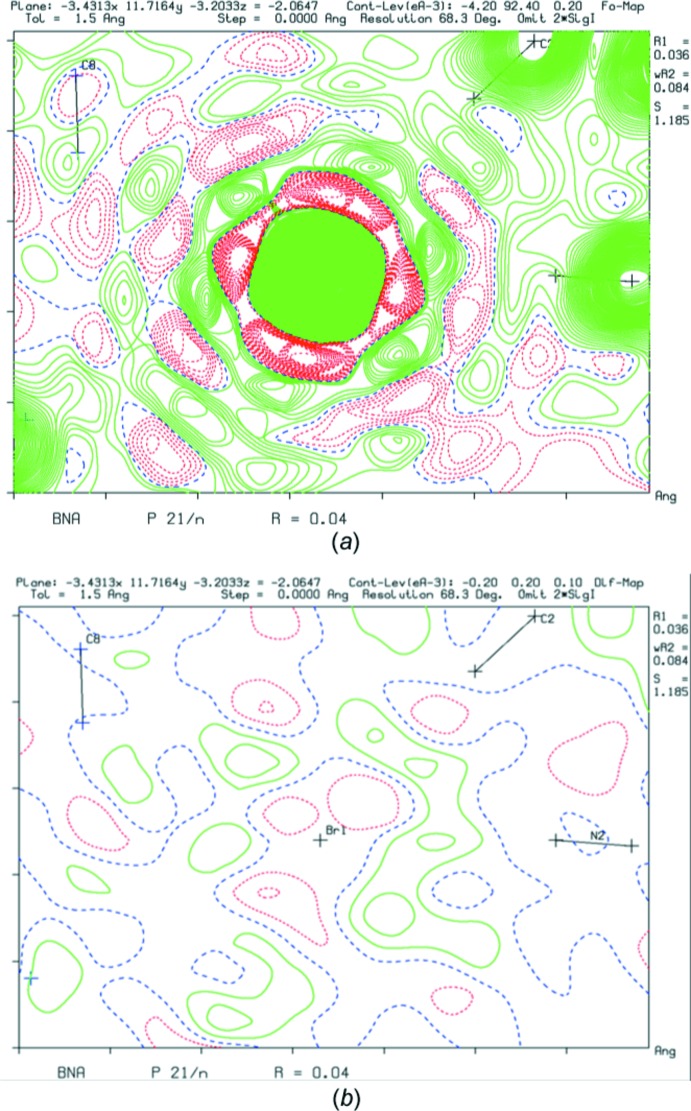
(*a*) Truncation ripples in an *F*_obs_ map and (*b*) an essentially clean associated difference-density map.

A special type of residual difference density concerns density near heavy atoms, usually within ∼1 Å. These are often artifacts related to improper correction for absorption and similar systematic effects. They should not be confused with or explained as arising from positive and negative ripples around heavy atoms, related to resolution truncation, as might be seen in *F*_obs_ maps (Fig. 4 ▸ *a*) but essentially absent in the associated difference-density map (Fig. 4 ▸ *b*). Absorption artifacts usually show up as a symmetrical pattern of positive and negative peaks around the heavy atoms, correlated with the external shape of the crystal.

**Figure 5:**
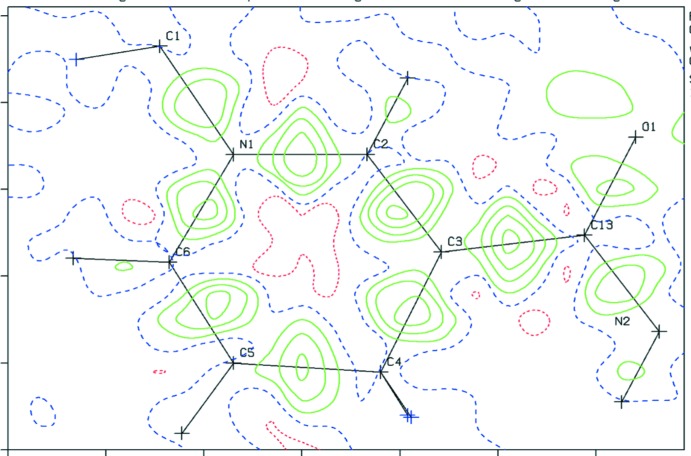
Difference-density map with residual density maxima on bonds for a good structure refined with spherical atomic scattering factors (AIM model). Contours are drawn with 0.1 e Å^−3^ inter­vals.

Structure reports for organic compounds based on good data and refined with the AIM model usually have their largest residual electron density on bonds (Fig. 5 ▸). Poor data result in noisy difference maps.

### Convergence quality criteria   

A low *R* value is frequently used as a criterion for a good structure. Unfortunately, that goal is often achieved in structure reports by limiting the resolution of the data used in the refinement or by removing outliers without proper justification. Other parameters such as the data-to-parameter ratio, *wR*_2_ and *S* values and weighting parameter values have to be considered as well. A refinement program such as *SHELXL* optimizes two parameter values in the reflection-weighting expression to achieve an *S* value close to 1.0. Failure to come close to that value might be indicative of unresolved issues (in the data or the model). In addition large values, in particular for the second parameter, in the *SHELXL* weighting expression may convey a similar message. A non-Gaussian error distribution of the observed and calculated intensities may be visualized with a normal probability plot (included in the *name.ckf* listing). The central part of the plot should be straight and plot not too S-shaped, indicating many outliers.

An even more detailed analysis can be obtained with the analysis-of-variance where the fit of the model with the data is analysed as a function of variables such as resolution and *F*_calc_/*F*_calc_(max). Significant deviations of the scaling *K* and GooF values from 1.0 may indicate improper integration issues and or structure-model errors. Details can be found in the *name.ckf* file for the associated ALERTS.

A sufficient reflection data (*N*) to refined parameter (*p*) ratio is expected. The number of reflections in this ratio is the number of Laue group averaged reflections. The *checkCIF* test criteria for the *N*:*p* ratio are set on values that can be met with good data for both centrosymmetric and non-centrosymmetric structures.

### Absolute structure   

Non-centrosymmetric structures are not always enanti­o­pure. A twinning model was introduced by Flack (1983 ▸) to take this into account with a refined Flack *x* parameter describing the mixture fraction. As many Bijvoet pairs as possible will be needed for a reliable refinement of that parameter. Usually, as a first step to establish a proper value for the Flack parameter, the non-centrosymmetric structure is refined without this refineable parameter included. Subsequently a post-refinement analysis is performed with the observed and calculated Bijvoet differences to estimate the value of the Flack parameter with associated estimated standard uncertainty. Various estimates for that are in use such as the Parsons *et al.* (2013 ▸) and Hooft *et al.* (2008 ▸, 2009 ▸, 2010 ▸) parameter values. Based on this analysis, subsequent refinement will be in order, including the Flack *x* parameter, when the estimated values deviate significantly from zero, including proper inversion of the coordinates when greater than 0.5. Criteria for determining valid absolute-structure assignments are give by Flack & Bernardinelli (2000 ▸).

## Validation   

Full validation with the program *PLATON* is done on both the *name.cif* file, containing the experimental and refinement data, and the *name.fcf* file, containing the final observed, calculated and σ values of the reflections used in the refinement. The *name.cif* file includes, where possible, both the detailed refinement instructions and the unmerged reflection data. A *name.cif* file created with a recent version of *SHELXL* includes all data necessary to recreate the final *name.fcf* file, thus eliminating the need to explicitly provide that file. The *name.fcf* file is used for a detailed analysis of the refinement. The result of that analysis is written to the *name.ckf* file. The ALERTS derived from the analysis of the *name.cif* and *name.fcf* data, based on criteria detailed in a file named *check.def*, are written to the *name.chk* file. By default, a built-in version of that file is used. *name.cif* files created with recent *SHELXL* versions include checksums meant to ensure the credibility of the embedded *name.res*, *name.hkl* and, in the case of a SQUEEZED structure, *name.fab* data as tested with the SHELX *shredcif* utility. Those embedded data should not be edited in order to avoid the IUCr/*checkCIF* requirement to supply the final *name.fcf* file.

Validation ALERTS should not be ignored or worked around. Level A ALERTS that cannot be resolved should be accompanied by a VRF record with an explanation why that ALERT can be ignored in the case at hand. However, it is even better to refer to some explanatory text in the report.

The CIF file to be validated may be a concatenation of one or more structural CIFs, each preceded by the data-block identifier *data_ID*, where *ID* should be distinct (*e.g*. I, II, *etc*.). An additional CIF-structured block *data_global* with non-structural information (*e.g.* journal information and publication text for *Acta Cryst. C* and *E*) can be included (preferably as the first block). Similarly, the associated FCF blocks (when not recreatable from the CIF file data), with identical *data_ID*, should be concatenated into one *name.fcf* file for *Acta Cryst*. submissions.

### Formula   

The expressions for the *sum formula* and *moiety formula* are well defined in the case of isolated mol­ecular species in general positions with associated *Z* and mol­ecular weight values, consistent with the provided chemical scheme. In cases where (some) of the atoms are located on special positions or with polymeric structures, things are less well defined. *checkCIF* offers a suggestion that may be overruled where more appropriate. The main requirement is consistency with associated data.

Situations where part of the structure remains uninter­preted in terms of explicit atomic parameters need special treatment. *PLATON*/*checkCIF* accepts the specification of information about the content of the ‘solvent’ voids to be specified between square brackets [], for example ‘[+solvent]’ when the nature of the solvent (mixture) is unclear or ‘[+toluene]’. The contribution within the square brackets is not included in the calculated mol­ecular weight and density as reported in the validation report.

### Hirshfeld test   

The Hirshfeld rigid-bond test (Hirshfeld, 1976 ▸) was originally not introduced as a validation test but turned out to be also useful as a validation tool. Organic mol­ecules were the focus of its original use. The notion is that the components of the displacement parameters of the two atoms in a covalent bond have approximately equal opposite values along that bond. This notion was extended to the generally weaker coordination bonds to a metal and transformed into a validation test. In this test, the Hirshfeld difference divided by its associated s.u. is compared with empirical threshold values. Large deviations may point to erroneously mis-assigned atom types. This test turned out to be instrumental in discovering published structures with deliberately changed atom types.

### Resonant scattering factors   

Refinement programs such as *SHELXL* include resonant scattering factor values (*f*′ and *f*′′) for most atom types and common X-ray sources (Cu *Kα*, Ag *K*α, Mo *K*α *etc*.). Values for other wavelengths (such as those for a synchrotron data collection) will need to be user supplied. The *f*′ and *f*′′ values found in the *name.cif* file are checked against those inter­polated from inter­nal tables in *PLATON. PLATON*/*checkCIF* uses data published by Brennan & Cowan (1992 ▸) for that purpose.

### Occupancy and multiplicity   

The *occupancy* factor as documented in the CIF is sometimes confused with the *site-occupancy factor* (SOF) as used in refinement programs such as *SHELXL* that include a weighting factor for atoms in special positions. For example, a SOF value of 0.5 for a fully occupied atom located at an inversion center should be reported with an *occupancy* of 1.0 in the CIF.

There are two, sometimes confusing, ways to specify the atom-site multiplicity associated with the occupation in the CIF: *_atom_site_site_symmetry_multiplicity (ssm)* and *atom_site_site_symmetry_order* (sso). Their relation is sso = nsym/ssm, where nsym = number of symmetry operations of the space group. *SHELXL* reports sso values in the CIF rather than the ssm values given in the *Inter­national Tables for Crystallography, Volume A.* SOF and occupancy are related by SOF = occupancy/sso.

### Short inter­molecular contacts   

Distances between atoms in the different mol­ecular species in a crystal structure are expected to be approximately equal to or greater than the sum of the van der Waals radii of the two atoms involved. *checkCIF* will flag cases where this distance is shorter.

Short H⋯H contacts may be related to hydrogen atoms placed in incorrect calculated positions. This might be due to a wrong hybridization assignment or putting the CH_3_ moiety in the wrong staggered or eclipsed orientation or not carrying out a rigid-rotating group refinement for the methyl group. Other not uncommon cases are the mis-assignment of a non-hydrogen atom carrying a hetero atom (*e.g*. S or N) and a C—H in a hetero ring moiety. Exchanging the assignments should solve the issue.

Short contacts involving disordered atom sites should be inspected in detail to ensure that they are physically possible and are not artefacts of the disorder model.

Short O⋯O or O⋯N contacts generally point to a missing hydrogen atom in this contact. This usually involves a smeared H atom in the middle of a short acid hydrogen bond, which may or may not be symmetrical (Fábry, 2018 ▸).

A valid exception concerns short halogen⋯halogen inter­actions (see *e.g*. Mukherjee & Desiraju, 2014 ▸). They are flagged as inter­esting information on the structure.

### FCF validation   

Details for reflection-related ALERTS in the *name.chk* file can be found in the CKF file (*name.ckf*). |*I*_obs_| − |*I*_calc_| outliers are listed along with information on missing reflections. The listing of the percentage of observed data with *I*_obs_ > 2 σ(*I*_obs_) as a function of sin (θ)/λ can be useful to optionally determine a noise cut-off level for reflections to be excluded from the refinement. The full *SHELXL-*style analysis-of-variance table is included for details of the associated ALERTS concerning deviations from expected values. Finally there are details listed for the check for twinning, absolute structure and a detailed analysis of the peaks in the final difference map.

### The unmerged reflection file   

An archived CIF is expected to include the unmerged reflection data on which a study is based. That file can be used to investigate unresolved issues concerning the correct space-group assignment. A merged reflection file has lost information on the quality and data consistency of systematic absences and indications for the lack of a centre of symmetry and absolute structure.

Unfortunately, the unmerged reflection file is already several steps away from the primary experimental data: the diffraction images. Data reduction (integration, correction for absorption *etc*.) is usually poorly documented and reported, being largely a black-box process using proprietary diffractometer software packages. Information about scattering effects outside the integrated diffraction spots and their shape is easily lost but often relevant to understand the origin of a poor structure refinement. The FAIR (findable, accessible, inter­operable and reusable; Helliwell, 2019 ▸) initiative addresses this issue.

A structure report can also be based on massaged or artificial data, as exemplified by data archived with the CSD. Those cases need not always to be fraudulent but rather related to poor chemical knowledge and inexperience with the available options in software packages. Software packages may include options to create artificial reflection data meant to be used for test purposes only.

*checkCIF* includes a number of checks that should flag issues with the data that need to be investigated in more detail. An example is the ALERT that reports that there is no residual density on bonds in the difference map of a ‘too good-looking’ structure (assuming refinement with spherical atomic scattering factors). A 1/σ(*I*_obs_) *versus* ^10^log(*I*_obs_) plot (Diederichs, 2010 ▸) or a σ(*I*_obs_) *versus* 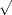
(*I*_obs_) plot may be useful to check for and illustrate the use of fabricated ‘experimental’ reflection data.

## A *PLATON*/*checkCIF* validation example   

**Figure 6:**
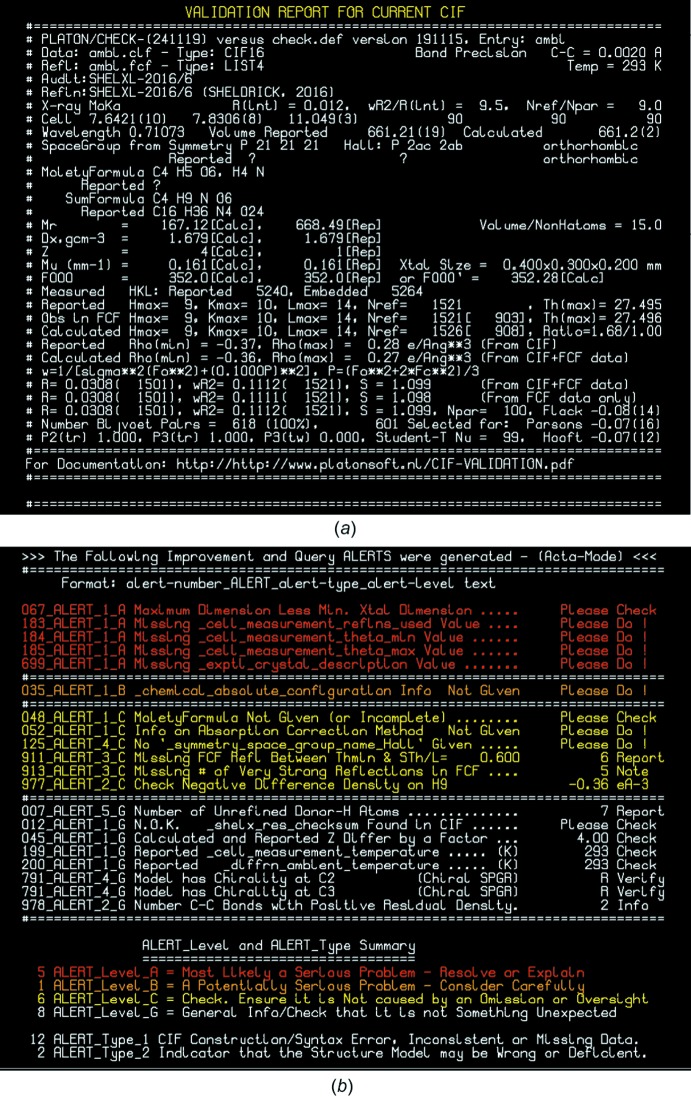
*PLATON*/*checkCIF* report: (*a*) structural data summary and (*b*) ALERT listing.

Fig. 6 ▸ illustrates a *PLATON*/*checkCIF* validation report, as obtained with a local *checkCIF* implementation, with a number of issues to be addressed. Fig. 6 ▸ *a* offers a summary of some relevant information such as experimental data and refinement results. The residual-density ranges, as reported in the *name.cif* file, are compared with those calculated by *PLATON* from the refined parameters in the *name.cif* file and the merged reflection data in the *name.fcf* file. Those values are expected to be closely identical within rounding errors. Three sets of *R*, *wR*_2_ and *S* values are calculated and compared. The first set is again calculated from the *name.cif* and *name.fcf* data. The second set is based on the observed and calculated reflection data in the *name.fcf* file, along with the weighting parameters taken from the *name.cif* file. The third set are the corresponding values as reported in the *name.cif* file. Where relevant, three types of ‘Flack parameter’ values are reported, being the Flack *x* value reported in the *name.cif* file, a *PLATON*-based estimate of the Parsons *z* value and a *PLATON*-based estimate of the Hooft *y* parameter value.

Fig. 6 ▸ *b* lists the ALERTS for this example. The A and B level ALERTS all refer to missing data and should be easy to address. So are the first three C level ALERTS. ALERT #977 indicates that atom H9 is located in a location not supported by the data. The #007 ALERT implies that the H-atom positions are not necessarily to be trusted for detailed hydrogen-bond analysis. ALERT #012 indicates that the embedded *name.res* file is corrupt or edited. A non-zero value of ALERT #978 can be taken as a measure of the quality of the difference density map of a refined structure (assuming that spherical atomic scattering factors were used).

## Concluding remarks   

*checkCIF* offers a machine-generated report in the form of ALERTS. It does not give a ‘good structure’ or ‘bad structure’ (or ’publish’ or ’reject’) verdict. That is left to human beings, either referees or users of the reported results. The value of a structure report lies in the added reliable scientific information. A well-documented poor-quality structure may be fine if supporting part of the reported chemistry in a chemical journal, but less suitable for publication in a crystallographic journal such as *Acta Crystallographica* that aims to publish the best attainable quality of the reported structure, the latter in view of future use of the data in unrelated research such as non-AIM model refinement.

Not all reported issues with a structure report can be resolved with access to the reflection data. Some can be resolved only with access to the diffraction images. Work on the archiving of those data is underway (Kroon-Batenburg *et al.*, 2017 ▸).

Current IUCr/*checkCIF* validation procedures mainly cover single-crystal structure reports refined with the atoms-in-mol­ecule (AIM) model. Refinements against powder diffraction data (*e.g. GSAS*; Toby & Von Dreele, 2013 ▸) and non-AIM model-based refinements [*e.g. IDEAL* (Lübben *et al.*, 2019 ▸) and *OLEX2*/*HARt* (Fugel *et al.*, 2018 ▸)] are currently only partly covered. The same applies to incommensurate structures (*JANA*; Petricek & Dusek, 2000 ▸) and structure determinations based on electron diffraction. Those will need the development of specialized validation procedures of the associated experimental, refinement and inter­pretation of the reported results.

## *checkCIF* software accessability   

The IUCr CIF/FCF validation facility is available through https://checkcif.iucr.org along with related tools.

A Microsoft Windows executable for local use is available from http://www.chem.gla.ac.uk/louis/software/platon.

Alternatively, a local copy of the source code with implementation instructions for LINUX and MacOS implementation of *PLATON*/*checkCIF* is available from http://www.platonsoft.nl/xraysoft. That version depends on an inter­face to the X-Windows system. A special version of the software is available without that dependency. Both the full and special versions of *PLATON* allow for easy keyboard instructions to run *checkCIF*. The keyboard instruction *platon -u name.cif* will produce a full *checkCIF* report on the files *name.chk* and *name.ckf*. The alternative command *platon -U name.cif* will not include the short explanations of the ALERTed-for issues.

## References

[bb1] Betteridge, P. W., Carruthers, J. R., Cooper, R. I., Prout, K. & Watkin, D. J. (2003). *J. Appl. Cryst.* **36**, 1487.

[bb2] Brennan, S. & Cowan, P. L. (1992). *Rev. Sci. Instrum.* **63**, 850–853.

[bb3] Burla, M. C., Camalli, M., Carrozzini, B., Cascarano, G. L., Giacovazzo, C., Polidori, G. & Spagna, R. (2003). *J. Appl. Cryst.* **36**, 1103. 10.1107/s0108767399007205 10927316

[bb4] Clegg, W. (2019). *Acta Cryst.* E**75**, 1812–1819. 10.1107/S2056989019014907 PMC6895943 31871736

[bb5] Cooper, R. I., Flack, H. D. & Watkin, D. J. (2017). *Acta Cryst.* C**73**, 845–853. 10.1107/S2053229617013304 29111508

[bb6] Diederichs, K. (2010). *Acta Cryst.* D**66**, 733–740. 10.1107/S0907444910014836 20516626

[bb7] Dolomanov, O. V., Bourhis, L. J., Gildea, R. J., Howard, J. A. K. & Puschmann, H. (2009). *J. Appl. Cryst.* **42**, 339–341.

[bb44] Fábry, J. (2018). *Acta Cryst.* E**74**, 1344–1357. 10.1107/S2056989018011544 PMC6127720 30225130

[bb8] Farrugia, L. J. (2012). *J. Appl. Cryst.* **45**, 849–854.

[bb43] Feast, G. C., Haestier, J., Page, L. W., Robertson, J., Thompson, A. L. & Watkin, D. J. (2009). *Acta Cryst* C**65**, o635–o638. 10.1107/S0108270109046952 19966448

[bb9] Flack, H. D. (1983). *Acta Cryst.* A**39**, 876–881.

[bb10] Flack, H. D. & Bernardinelli, G. (2000). *J. Appl. Cryst.* **33**, 1143–1148.

[bb11] Fugel, M., Jayatilaka, D., Hupf, E., Overgaard, J., Hathwar, V. R., Macchi, P., Turner, M. J., Howard, J. A. K., Dolomanov, O. V., Puschmann, H., Iversen, B. B., Bürgi, H.-B. & Grabowsky, S. (2018). *IUCrJ*, **5**, 32–44. 10.1107/S2052252517015548 PMC5755575 29354269

[bb12] Groom, C. R., Bruno, I. J., Lightfoot, M. P. & Ward, S. C. (2016). *Acta Cryst.* B**72**, 171–179. 10.1107/S2052520616003954 PMC4822653 27048719

[bb13] Hall, S. R. (1981). *Acta Cryst.* A**37**, 517–525.

[bb14] Hall, S. R., Allen, F. H. & Brown, I. D. (1991). *Acta Cryst.* A**47**, 655–685.

[bb15] Helliwell, J. R. (2019). *Struct. Dyn.* **6**, 054306. doi: 10.1063/1.5124439. eCollection 2019 Sep. 10.1063/1.5124439 PMC6816445 31673568

[bb16] Hirshfeld, F. L. (1976). *Acta Cryst.* A**32**, 239–244.

[bb17] Hooft, R. W. W., Straver, L. H. & Spek, A. L. (2008). *J. Appl. Cryst.* **41**, 96–103. 10.1107/S0021889807059870 PMC2467520 19461838

[bb18] Hooft, R. W. W., Straver, L. H. & Spek, A. L. (2009). *Acta Cryst.* A**65**, 319–321. 10.1107/S0108767309009908 19535853

[bb19] Hooft, R. W. W., Straver, L. H. & Spek, A. L. (2010). *J. Appl. Cryst.* **43**, 665–668. 10.1107/S0021889807059870 PMC2467520 19461838

[bb20] Immirzi, A. (2009). *J. Appl. Cryst.* **42**, 362–364. 10.1107/S0021889808044142 PMC3246815 22477768

[bb21] Johnson, C. K. (1976). *ORTEPII*. Report ORNL -5138. Oak Ridge National Laboratory, Tennessee, USA.

[bb22] Kroon-Batenburg, L. M. J., Helliwell, J. R., McMahon, B. & Terwilliger, T. C. (2017). *IUCrJ*, **4**, 87–99. 10.1107/S2052252516018315 PMC5331468 28250944

[bb23] Le Page, Y. (1987). *J. Appl. Cryst.* **20**, 264–269.

[bb24] Le Page, Y. (1988). *J. Appl. Cryst.* **21**, 983–984.

[bb25] Lübben, J., Wandtke, C. M., Hübschle, C. B., Ruf, M., Sheldrick, G. M. & Dittrich, B. (2019). *Acta Cryst.* A**75**, 50–62. 10.1107/S2053273318013840 PMC6302932 30575583

[bb26] Marsh, R. E. & Spek, A. L. (2001). *Acta Cryst.* B**57**, 800–805. 10.1107/S0108768101014331 11717479

[bb27] Mukherjee, A. & Desiraju, G. R. (2014). *IUCrJ*, **1**, 49–60. 10.1107/S2052252513025657 PMC4104968 25075319

[bb28] Müller, P., Herbst-Irmer, R., Spek, A. L., Schneider, T. R. & Sawaya, M. R. (2006). *Crystal Structure Refinement*. Oxford University Press.

[bb29] Palatinus, L. & Chapuis, G. (2007). *J. Appl. Cryst.* **40**, 786–790.

[bb30] Parsons, S., Flack, H. D. & Wagner, T. (2013). *Acta Cryst.* B**69**, 249–259. 10.1107/S2052519213010014 PMC3661305 23719469

[bb31] Petricek, V. & Dusek, M. (2000). *JANA2000*. Institute of Physics, Czech Academy of Sciences, Prague, Czech Republic.

[bb32] Sheldrick, G. M. (2008). *Acta Cryst.* A**64**, 112–122. 10.1107/S0108767307043930 18156677

[bb33] Sheldrick, G. M. (2015*a*). *Acta Cryst.* A**71**, 3–8.

[bb34] Sheldrick, G. M. (2015*b*). *Acta Cryst.* C**71**, 3–8.

[bb35] Sluis, P. van der & Spek, A. L. (1990). *Acta Cryst.* A**46**, 194–201.

[bb36] Spek, A. L. (2003). *J. Appl. Cryst.* **36**, 7–13.

[bb37] Spek, A. L. (2009). *Acta Cryst.* D**65**, 148–155. 10.1107/S090744490804362X PMC2631630 19171970

[bb38] Spek, A. L. (2015). *Acta Cryst.* C**71**, 9–18. 10.1107/S2053229614024929 25567569

[bb39] Spek, A. L. (2018). *Inorg. Chim. Acta*, **470**, 232–237.

[bb40] Thompson, A. L. (2019). *Crystallogr. Rev.* **25**, 3–53.

[bb41] Toby, B. H. & Von Dreele, R. B. (2013). *J. Appl. Cryst.* **46**, 544–549.

